# Antinephrin-Associated Primary Focal Segmental Glomerulosclerosis Successfully Treated With Plasmapheresis

**DOI:** 10.1016/j.ekir.2024.06.038

**Published:** 2024-07-01

**Authors:** Iain Bressendorff, Karl Emil Nelveg-Kristensen, Maryam Ghasemi, Andrew J.B. Watts, Johanna Elversang, Keith H. Keller, Finn Cilius Nielsen, Wladimir Szpirt, Astrid Weins

**Affiliations:** 1 Department of Nephrology, Rigshospitalet, Copenhagen, Denmark; 2 Renal Division, Brigham and Women’s Hospital, Harvard Medical School, Boston, Massachusetts, USA; 3 Department of Pathology, Rigshospitalet, Copenhagen, Denmark; 4 Broad Institute of Harvard and Massachusetts Institute of Technology, Cambridge, Massachusetts, USA; 5 Center for Genomic Medicine, Rigshospitalet, Copenhagen, Denmark

## Introduction

Minimal change disease and primary focal segmental glomerulosclerosis (FSGS) have similar clinical manifestations, both presenting with rapid-onset nephrotic syndrome and often amendable to treatment with high-dose glucocorticoids. Recently, antinephrin antibodies (Ab) have been reported to be present in a subset of patients with minimal change disease as well as in patients with primary FSGS with recurrent massive proteinuria after kidney transplantation.^1^, ^2^, ^3^ Infusion of antinephrin Ab in mice results in rapid-onset proteinuria and subsequent FSGS lesions.^4^ These findings suggest that antinephrin Ab may be a possible causative permeability factor in some cases of minimal change disease and primary FSGS. Here, we present a case of severe primary FSGS with high levels of antinephrin Ab in plasma, which was successfully treated with plasmapheresis (PLEX). Key teaching points of this case are presented in Table 1.

**Table 1:** Teaching points

Antinephrin antibodies have been reported in minimal change disease and recurrent FSGS after kidney transplantation. Here, we report the first case of antinephrin positive primary FSGS.
Disappearance of antinephrin antibodies had a temporal association with disease remission.
Plasmapheresis may be a useful adjunct to high-dose glucocorticoids in severe or treatment-resistant antinephrin-positive primary FSGS.

FSGS, focal segmental glomerulosclerosis.

## Case Presentation

**Figure 1:**
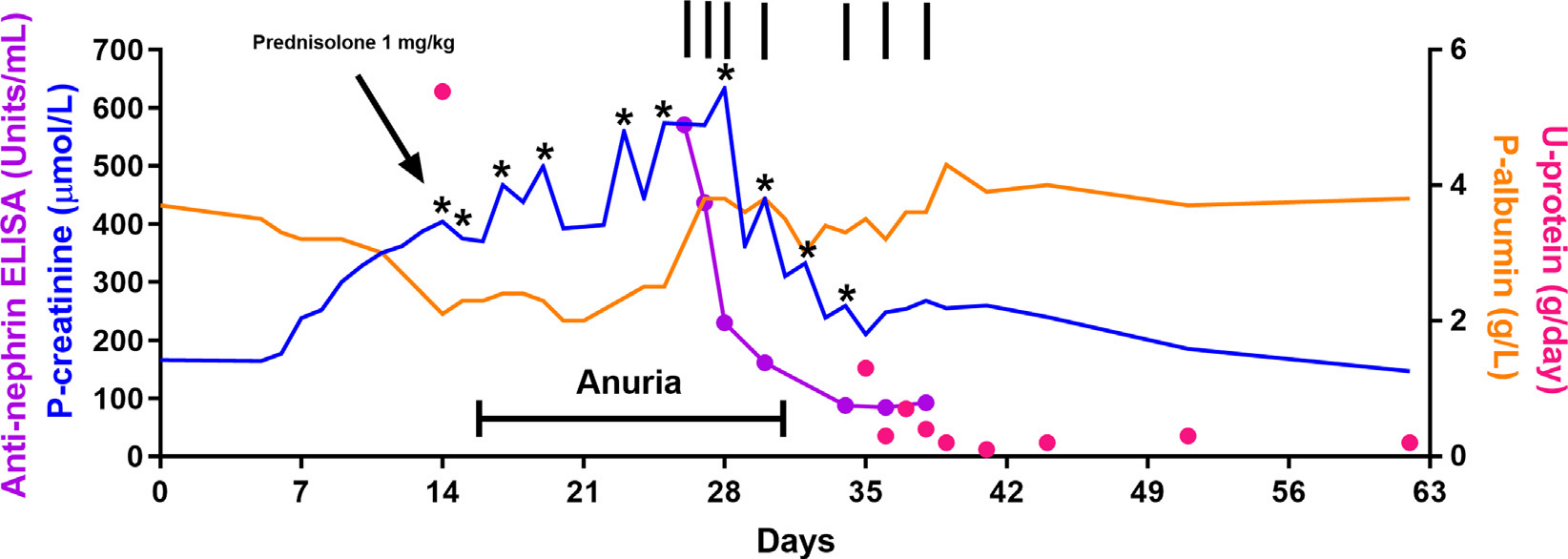
Time course of disease and treatment. Asterix represents hemodialysis sessions, vertical lines represent plasmapheresis sessions. HD, hemodialysis; PLEX, plasmapheresis.

An 84-year-old man with previous medical history of stable coronary artery disease, atrial fibrillation, hypertension, hyperlipidemia, stage 3 chronic kidney disease (estimated glomerular filtration rate of 45 ml/min per 1.73 m^2^ without proteinuria), peripheral arterial disease and asthma, presented to an outside hospital with shortness of breath and edema, which had developed over 1 week. He was started on a loop diuretic and discharged from hospital 2 days later. Urine tests were not performed. Three days later, the patient again presented to the same hospital with worsening shortness of breath and edema, and now had progressive acute kidney injury with hypoalbuminemia (Figure 1). A urine dipstick showed +3 for albumin, and proteinuria was later quantified as 5.2 g/d on a 24-hour urine collection. His acute kidney injury progressed during the following week, and 15 days after the initial presentation he became completely anuric. A kidney ultrasound showed 2 normal-sized kidneys without hydronephrosis. The patient was transferred to the Department of Nephrology, Rigshospitalet, Denmark, and upon arrival he was initiated on oral prednisolone 1 mg/kg and started on hemodialysis. A kidney biopsy was performed 3 days later (delayed due to oral anticoagulation), which showed FSGS in 1 out of 13 glomeruli (2 of which were globally sclerosed) and approximately 10% interstitial fibrosis and tubular atrophy (Supplementary Methods and Supplementary Figure S1). There were no signs of acute tubular necrosis. Immunofluorescence revealed punctate IgG “dusting” with overlap between IgG and nephrin, as previously described,^1^ but was otherwise negative for IgM, IgA, C3, C1q, as well as kappa and lambda light chains. Electron microscopy revealed diffuse foot process effacement at >90%.

The clinical presentation of nephrotic syndrome with progressive acute kidney injury and a kidney biopsy showing FSGS was suspicious for primary (permeability factor related) FSGS. After 14 days of high-dose glucocorticoids, the patient showed no signs of improvement and was still completely anuric. In an attempt to remove any circulating pathogenic factor(s), treatment with PLEX was initiated. Blood samples were drawn prior to each PLEX treatment, which were later found to be positive for antinephrin Ab by enzyme-linked immunosorbent assay, levels of which declined progressively with each PLEX treatment (Supplementary Methods and Supplementary Figure S2). After the fourth PLEX session, antinephrin Ab levels were below the threshold previously defined for positivity based on a healthy control population without kidney disease.^1^ At the same time, urine output increased, and plasma creatinine declined. The patient completed 7 sessions of PLEX with complete resolution of his nephrotic syndrome and acute kidney injury. Kidney function returned to baseline and over the following months, the dose of prednisolone was tapered and mycophenolate mofetil was added for maintenance. Three months after clinical remission, plasma antinephrin Ab was still below the limit of detection. Whole-genome sequencing for risk variants did not identify any known or likely pathogenic variants in known FSGS risk genes. A year and a half after presentation, kidney function is stable without proteinuria and the patient is treated with mycophenolate mofetil only as maintenance therapy.

## Discussion

We present the first case of antinephrin-positive primary FSGS, which achieved complete clinical and immunological remission in temporal association with the disappearance of antinephrin Ab. However, given the observational nature of this case report, causation between antinephrin Ab and the patient’s nephrotic syndrome cannot be definitively concluded. In addition, we note the temporal association of PLEX initiation with reduction of antinephrin Ab. Again, no causality can be inferred from this case report because although there was seemingly no effect of high-dose prednisolone after 2 weeks of treatment, it is possible that production of antinephrin Ab had already been shut off by the prednisolone treatment. Therefore, levels of antinephrin would perhaps have decreased even without PLEX. It does seem likely, however, that PLEX would hasten the removal of antinephrin Ab, akin to how PLEX removes autoantibodies in other autoimmune kidney diseases (e.g., antiglomerular basement membrane disease), and removal of antinephrin Ab by PLEX may therefore be useful as an adjunct to high-dose glucocorticoids in cases of severe antinephrin–associated primary FSGS. Further research into the role of antinephrin Ab and therapies targeting these in antinephrin Ab-positive nephrotic syndrome, is warranted.

## Disclosure

AJBW, KK, and AW report a patent pending for an antinephrin antibody enzyme-linked immunosorbent assay. All the other authors declared no competing interests.

## Patient Consent

The authors declare that they have obtained consent from the patient discussed in the report.

## Supplementary Material

Supplementary File (PDF) **Supplementary Methods. Supplementary Materials. Figure S1.** Kidney biopsy evaluation. **Figure S2.** Levels of antinephrin in plasma by enzyme-linked immunosorbent assay measured immediately prior to each session of plasmapheresis.

## Footnotes

Supplementary File (PDF)

**Supplementary Methods.**

**Supplementary Materials.**
**Figure S1.** Kidney biopsy evaluation. **Figure S2.** Levels of antinephrin in plasma by enzyme-linked immunosorbent assay measured immediately prior to each session of plasmapheresis.
