# Supplementary material for: Antinephrin-Associated Primary Focal Segmental Glomerulosclerosis Successfully Treated With Plasmapheresis
Source: Kidney Int Rep. 2024 Jul 1;9(9):2829–31. doi: 10.1016/j.ekir.2024.06.038 (PMC11403026; doi:10.1016/j.ekir.2024.06.038)
Supplement: Supplementary File (PDF) — Supplementary Methods. Supplementary Materials. Figure S1. Kidney biopsy evaluation. Figure S2. Levels of antinephrin in plasma by enzyme-linked immunosorbent assay measured immediately prior to each session of plasmapheresis. [file mmc1.pdf]

Anti-nephrin-associated primary FSGS successfully treated with plasmapheresis

Supplementary Methods and Materials

Circulating anti-nephrin Ab were measured by ELISA and confocal microscopy was used to co-localize IgG and nephrin in kidney biopsy tissue, as previously described (Watts et al, JASN 2022). The threshold for disease activity was derived from a population of healthy controls.

The patient's DNA was analyzed by PCR-free whole genome sequencing (WGS) on an Illumina NextSeq providing 10 times (10X) coverage of more than 95% of the human genome and a median coverage of 30X. In order to examine the presence of putative FSGS associated variants the following gene panel was employed: *ACTN4, APOL1, ANLN, ARHGAP24, COL4A3, COL4A4, COL4A5, COL4A6, INF2, LMX1B, MAFB, MYH9, NFKB2, PAX2, TRIM8, TRPC6, WT1, ANKFY1, ARHGDIA, AVIL, CDK20, CD2AP, COQ2, COQ6, COQ8B/ADCK4, CRB2, CUBN, DAAM2, DGKE, DLC1, EMP2, FAT1, GAPVD1, GON7, ITGA3, ITGB4, ITSN1, ITSN2, KANK1, KANK2, KANK4, KAT2B, KIRREL1, LAMA5, LAGE3, LAMB2, MAGI2, MYO1E, NEU1, NPHS1, NPHS2, NUP107, NUP133, NUP160, NUP205, NUP93, OSGEP, PDSS2, PLCE1, PTPRO, SCARB2, SGPL1, SMARCAL1, TBC1D8B, TNS2, TP53RK, TPRKB, TTC21B, WDR4, WDR73, XPO5,* and *YRDC*.

Supplementary Figure S1 – Kidney biopsy evaluation

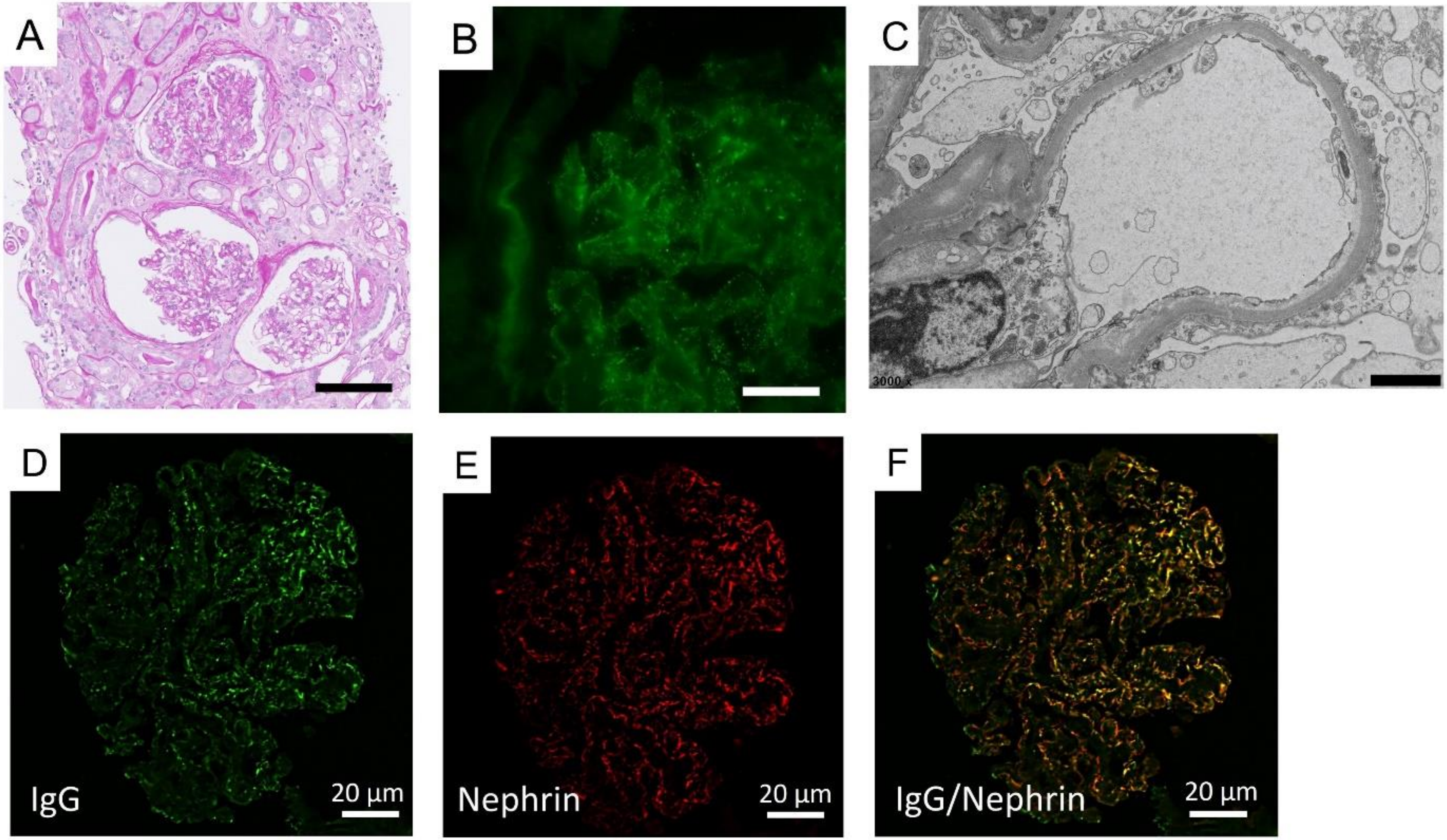

Supplementary Figure S1 – Kidney biopsy evaluation

Periodic Acid Schiff (PAS) staining reveals glomeruli with tip lesion FSGS, characterized by tuft adhesions around the tubular pole (A). Scale bar: 50 µm.

Immunofluorescence (IF) imaging for IgG shows global and diffuse punctate podocyte "dusting" for IgG (B). Scale bar: 20 µm.

Electron Microscopy (EM) Microscopy reveals diffuse podocyte foot process effacement in the absence of any electron dense deposits. Scale bar: 2 µm.

Confocal microscopy of a glomerulus stained for IgG (green) (D) and nephrin (red) (E) shows overlapping staining in a punctate pattern (F), as previously described in Watts et al, JASN 2022.

Supplementary Figure S2 – Levels of anti-nephrin in plasma by ELISA measured immediately prior to each session of plasmapheresis.

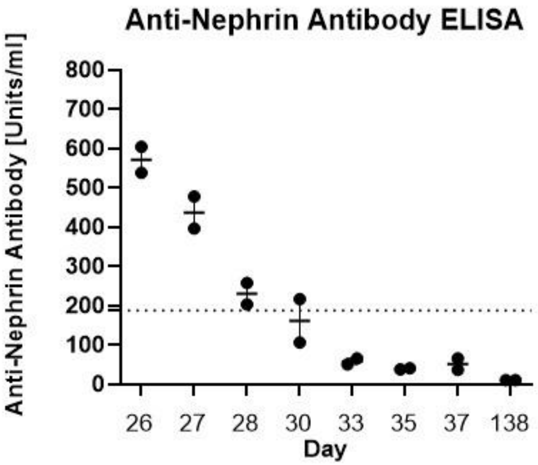

The threshold for disease activity is displayed as horizontal dotted line. Error bars represent standard error of the mean (SEM).
